# Molecular characterization of *Cryptosporidium* spp. and *Giardia duodenalis* in children in Egypt

**DOI:** 10.1186/s13071-018-2981-7

**Published:** 2018-07-11

**Authors:** Doaa Naguib, Adel H. El-Gohary, Dawn Roellig, Amro A. Mohamed, Nagah Arafat, Yuanfei Wang, Yaoyu Feng, Lihua Xiao

**Affiliations:** 1 Department of Hygiene and Zoonoses, Faculty of Veterinary Medicine, Mansoura University, Mansoura, 35516 Egypt; 2 Division of Foodborne, Waterborne, and Environmental Diseases, National Center for Emerging and Zoonotic Infectious Diseases, Centers for Disease Control and Prevention, Atlanta, GA 30329 USA; 3 Department of Poultry Diseases, Faculty of Veterinary Medicine, Mansoura University, Mansoura, 35516 Egypt; 4 Key Laboratory of Zoonosis of Ministry of Agriculture, College of Veterinary Medicine, South China Agricultural University, Guangzhou, 510642 China

**Keywords:** *Cryptosporidium*, *Giardia duodenalis*, Children, Egypt, Epidemiology, Subtypes

## Abstract

**Background:**

The transmission of *Cryptosporidium* spp. and *Giardia duodenalis* into humans varies according to species/genotypes of the pathogens. Although infections with both parasites are recorded in Egypt, few data are available on the distribution of *Cryptosporidium* species and *G. duodenalis* genotypes. The present study assessed the occurrence and genetic diversity of *Cryptosporidium* spp. and *G. duodenalis* in Egyptian children.

**Methods:**

In the present study, 585 fecal specimens were collected from children eight years old and younger in three provinces (El-Dakahlia, El-Gharbia and Damietta) during March 2015 to April 2016. PCR-RFLP analysis of the small subunit rRNA gene and sequence analysis of the 60 kDa glycoprotein gene were used to detect and subtype *Cryptosporidium* spp., respectively, whereas PCR and sequence analyses of the triose phosphate isomerase, glutamate dehydrogenase and β-giardin genes were used to detect and genotype *Giardia duodenalis*.

**Results:**

The overall infection rates of *Cryptosporidium* spp. and *G. duodenalis* were 1.4% and 11.3%, respectively. The *Cryptosporidium* species identified included *C. hominis* and *C. parvum*, each with three subtype families. The *C. hominis* subtypes were IbA6G3 (*n* = 2), IdA17 (*n* = 1), IdA24 (*n* = 1) and IfA14G1R5 (*n* = 1), while *C. parvum* subtypes were IIdA20G1 (*n* = 1), IIaA15G2R1 (*n* = 1), and IIcA5G3a (*n* = 1). The *G. duodenalis* identified included both assemblages A (*n* = 31) and B (*n* = 34). All *G. duodenalis* assemblage A belonged to the anthroponotic sub-assemblage AII, while a high genetic heterogeneity was seen within assemblage B.

**Conclusions:**

Data from this study are useful in our understanding of the genetic diversity of *Cryptosporidium* spp. and *G. duodenalis* in Egypt and the potential importance of anthroponotic transmission in the epidemiology of both pathogens.

**Electronic supplementary material:**

The online version of this article (10.1186/s13071-018-2981-7) contains supplementary material, which is available to authorized users.

## Background

Diarrhea is a worldwide public health issue, responsible for 2.3 billion sicknesses and 1.3 million deaths in 2015. It is the second most important cause of death among children under 5 years of age [1]. Most of the deaths are recorded in developing countries, particularly African countries. Various gastrointestinal pathogens, including bacteria, viruses and parasites cause diarrhea. Among the latter, *Cryptosporidium* spp. and *Giardia duodenalis* are common etiological agents in humans and animals globally [2, 3]. *Cryptosporidium* is second only to rotavirus in causing diarrhea and death in children in developing countries, responsible for 2.9 million cases annually in children aged < 24 months in the sub-Saharan Africa [4, 5]. Similarly, *G. duodenalis* is responsible for ~280 million cases of intestinal diseases per year worldwide [6]. *Cryptosporidium* spp. and *G. duodenalis* are transmitted in humans through the fecal-oral route, either directly by person-to-person transmission or contact with infected animals or indirectly *via* food-borne or water-borne transmission following ingestion of contaminated food or water [2, 3].

Currently, over 30 *Cryptosporidium* species have been recognized, but humans are mostly infected with *C. parvum* and *C. hominis* [7] with the former mostly transmitted anthroponotically while the latter can be transmitted either anthroponotically or zoonotically [8]. Similarly, among the eight established *G. duodenalis* genotypes (frequently referred as assemblages) identified using molecular tools, assemblages A and B are responsible for most human infections. Between them, assemblage A is also commonly seen in animals and thus could be responsible for zoonotic *G. duodenalis* infection [8, 9].

It has been noted that some subtype families of *C. parvum* are more frequently found in certain host species, such as IIa in cattle, IIc in humans, and IId in sheep and goats. While all three subtype families of *C. parvum* can infect humans, their distribution in humans differs geographically and socioeconomically, probably as a result of differences in the importance of various transmission routes [8]. Similarly, host adaptation also occurs within *G. duodenalis* assemblage A, with AI subtypes being more commonly found in domestic animals, AII subtypes mostly in humans, and AIII subtypes almost exclusively in wild ruminants [8, 9]. Thus, molecular characterizations of *Cryptosporidium* spp. and *G. duodenalis* at species and subtype levels are helpful in improving our understanding of cryptosporidiosis and giardiasis epidemiology [7].

Compared with other countries, few data exist on the occurrence of *Cryptosporidium* and *G. duodenalis* genotypes and subtypes in humans in Egypt. Previous microscopic and serologic studies had shown a common occurrence of *Cryptosporidium* spp. and *G. duodenalis* in humans in the country [10–12]. Only a few studies have examined the molecular characteristics of *Cryptosporidium* spp. and *G. duodenalis* in a small number of human clinical specimens [13–18]. The current study was conducted to collect data on the distribution of *Cryptosporidium* and *G. duodenalis* genotypes and subtypes in kindergarten age children (≤ 8 years) in order to improve our understanding of the transmission of these parasites in Egypt.

## Methods

### Specimen collection

**Fig. 1:**
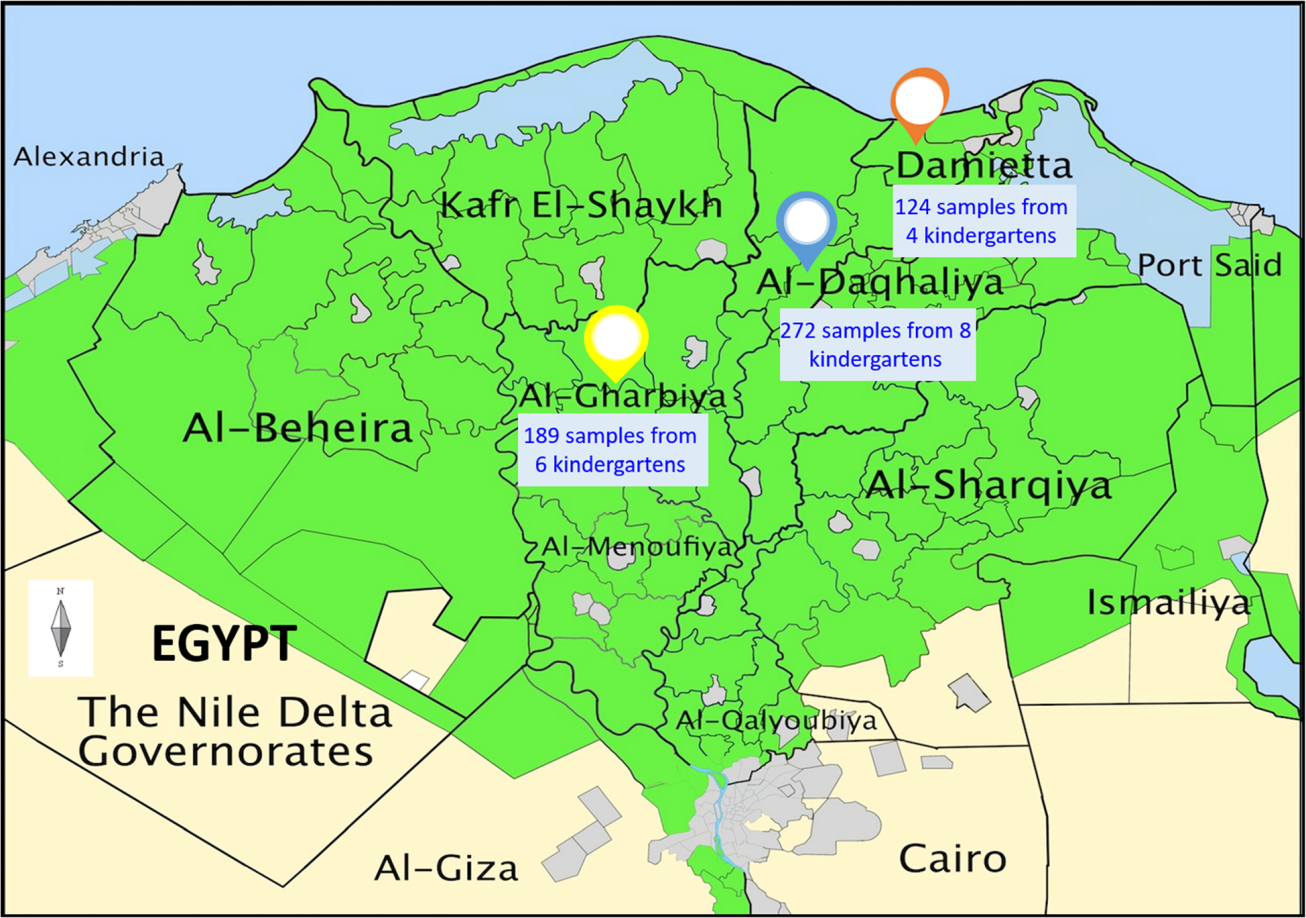
Map of Egypt showing the locations of study sites: El-Dakahlia, El-Gharbia and Damietta provinces

This study was conducted during March 2015 to April 2016 in El-Dakahlia, El-Gharbia, and Damietta provinces, Egypt (Fig. 1). Fresh stool specimens were collected monthly from 585 different children in 18 childcare centers, who ranged 2 to 8 years in age (median age: 4 years). These specimens were collected individually in sterile plastic cups and transported to the laboratory in coolers. Information on the age, gender, diarrhea and health status, animal contact and residency, was recorded from parents or guardians. Specimens were preserved in 70% ethanol and kept at 4 °C to prevent DNA deterioration prior to DNA extraction at the Centers for Disease Control and Prevention, Atlanta, GA, USA. No microscopy of pathogens was conducted during the study. Informed consent was obtained from the parents or guardians of the study children.

### DNA extraction

Stored stool specimens were washed twice with distilled water by centrifugation to remove ethanol. DNA was extracted from washed fecal materials using the FastDNA SPIN Kit for Soil (MP Biomedicals, Irvine, CA, USA) and manufacturer-recommended procedures. DNA was eluted in 100 μl molecular grade water and stored at -20 °C prior to molecular analyses.

### *Cryptosporidium* detection, genotyping and subtyping

All specimens were examined for *Cryptosporidium* spp. using a nested polymerase chain reaction (PCR) targeting a ∼834 bp fragment of the small subunit rRNA (*SSU rRNA*) gene [19]. *C. parvum-* and *C. hominis-*positive specimens were further analyzed by a nested PCR targeting a ∼850 bp fragment of the 60 kDa glycoprotein (*gp60*) gene [20]. Each analysis was conducted in duplicate, using *C. baileyi* and *C. parvum* DNA as the positive control for *SSU rRNA* and *gp60* PCR, respectively, and reagent-grade water as the negative control. *Cryptosporidium* species in the positive specimens were identified by RFLP analysis of the secondary *SSU rRNA* PCR products using restriction enzymes *Ssp*I (New England BioLabs, Ipswich, MA, USA) and *Vsp*I (Promega, Madison, WI, USA) as described [19]. *C. hominis* and *C. parvum* subtypes were identified by bidirectional DNA sequence analysis of the secondary PCR products of the *gp60* gene [20].

### *Giardia* detection, genotyping and subtyping

All 585 specimens were analyzed for *G. duodenalis* using nested-PCR assays targeting 3 genetic loci, including triose phosphate isomerase (*tpi*) [21], beta-giardin (*bg*) [22] and glutamate dehydrogenase (*gdh*) [23] genes. Specimens were identified as *G. duodenalis*-positive when the expected PCR product was obtained from at minimum one of the three loci. *G. duodenalis* genotypes and subtypes were identified by bidirectional DNA sequence analysis of the secondary PCR products.

### DNA sequence analyses

All positive secondary PCR products generated in the study were purified using Montage PCR filters (Millipore, Bedford, MA, USA) and sequenced in both directions on an ABI 3130 Genetic Analyzer (Applied Biosystems, Foster City, CA, USA). Nucleotide sequences generated were edited and assembled using the ChromasPro software (www.technelysium.com.au/ChromasPro.html). They were aligned against each other and reference sequences [7, 9] using ClustalX software (http://www.clustal.org/) to identify *Cryptosporidium* subtypes and *G. duodenalis* assemblages and subtypes. Multilocus genotypes (MLGs) of *G. duodenalis* assemblage A were identified based on nucleotide sequences at the *tpi*, *bg*, and *gdh* loci, using the established nomenclature system [9].

### Statistical analysis

The Chi-square test was used to compare *Cryptosporidium* and *G. duodenalis* infection rates between age groups (≤ 3 to 8 years), gender (boys and girls), residency (urban and rural), and children with and without gastrointestinal symptoms (diarrhea and abdominal pain) or animal contact (with and without). The relationship between age and diarrhea was assessed using the nonparametric Kendall’s tau_b and Spearman’s rho tests. The statistical analysis was performed using the SPSS software version 20.0 (IBM, Armonk, NY, USA). Differences were considered significant at *P* < 0.05.

## Results

### Occurrence of *Cryptosporidium* spp. and *G. duodenalis*

Of the 585 fecal specimens examined in this study from kindergarten children, 8 (1.4%) and 66 (11.3%) were positive for *Cryptosporidium* spp. and *G. duodenalis*, respectively. No concurrence of the two pathogens was detected in any of the specimens.

By age, the highest rates of *Cryptosporidium* (2.7%) and *G. duodenalis* (14.2%) infections were detected in children of age ≤ 3 years and 4 years, respectively; neither *Cryptosporidium* nor *G. duodenalis* were detected in children of 8 years in age (Table 1). The infection rates of both protozoans were similar between girls and boys (1.0% and 1.7% for *Cryptosporidium* and 11.1% and 11.5% for *G. duodenalis*, respectively) (*χ*^2^ = 0.460, *P* = 0.49 and *χ*^2^ = 0.011, *P* = 0.91, respectively).

**Table 1:** Occurrence of *Cryptosporidium* spp. and *Giardia duodenalis* in children by age, gender, diarrhea or abdominal pain occurrence, animal contact, residency and locality

Variable	No. of samples	No. of positive (%)					
		*Cryptosporidium* spp.	95% confidence interval		*Giardia duodenalis*	95% confidence interval	
			Lower limit	Upper limit		Lower limit	Upper limit
Age							
≤ 3 years	74	2 (2.7)	-0.009	0.063	7 (9.5)	0.028	0.161
4 years	141	3 (2.1)	-0.002	0.044	20 (14.2)	0.084	0.199
5 years	190	1 (0.5)	-0.005	0.015	26 (13.7)	0.088	0.185
6 years	136	2 (1.5)	-0.005	0.035	10 (7.4)	0.030	0.117
7years	27	0 (0.0)	0.000	0.000	3 (11.1)	-0.007	0.229
8 yeas	17	0 (0.0)	0.000	0.000	0 (0.0)	0.000	0.000
Gender							
Female	289	3 (1.0)	-0.001	0.021	32 (11.1)	0.074	0.147
Male	296	5 (1.7)	0.002	0.031	34 (11.5)	0.078	0.151
Diarrhea occurrence							
Yes	89	2 (2.3)	-0.008	0.054	17 (19.1)^a^	0.109	0.272
No	496	6 (1.2)	0.002	0.021	49 (9.9)^a^	0.72	0.125
Abdominal pain							
Yes	351	7 (2.0)	0.005	0.034	38 (10.8)	0.075	0.140
No	234	1 (0.4)	-0.004	0.012	28 (12.0)	0.078	0.161
Animal contact							
With	257	3 (1.2)	-0.001	0.025	27 (10.5)	0.067	0.142
Without	328	5 (1.5)	0.001	0.028	39 (11.9)	0.083	0.154
Residency							
Rural	332	5 (1.5)	0.001	0.028	40 (12.1)	0.085	0.156
Urban	253	3 (1.2)	-0.001	0.025	26 (10.3)	0.065	0.140
Locality							
El-Dakahlia	272	5 (1.8)	0.002	0.033	31 (11.4)	0.076	0.151
El-Gharbia	189	2 (1.1)	-0.003	0.025	24 (12.7)	0.079	0.174
Damietta	124	1 (0.8)	-0.007	0.023	11 (8.9)	0.038	0.139

^a^The difference between the two groups is significant

*Cryptosporidium* infection rate was 2.3% and 1.2 % in children with and without diarrhea, respectively (*χ*^2^ = 0.576, *P* = 0.44). In contrast, the infection rate of *G. duodenalis* was significantly higher in diarrheic children (19.1%) than in non-diarrheic ones (9.9%) (*χ*^2^ = 6.149, *P* = 0.01). There was also an insignificantly higher occurrence of *Cryptosporidium* spp. in children with abdominal pain (2.0%) than those without it (0.4%) (*χ*^2^ = 2.612, *P* = 0.10). In contrast, *G. duodenalis* infection rates were similar between the two groups (10.8% and 12.0%, respectively; *χ*^2^ = 0.134, *P* = 0.71). The infection rates of *Cryptosporidium* and *G. duodenalis* were similar between children with (1.2% and 10.5%, respectively) and without (1.5% and 11.9%, respectively) animal contact (*χ*^2^ = 0.146, *P* = 0.92 and *χ*^2^ = 0.128, *P* = 0.93, respectively). In addition, children in rural areas had *Cryptosporidium* and *G. duodenalis* infection rates (1.5% and 12.1%, respectively) similar to those in urban areas (1.2% and 10.3%, respectively; *χ*^2^ = 0.091, *P* = 0.76 and *χ*^2^ = 0.339, *P* = 0.56, respectively; Table 1). The infection rate of *Cryptosporidium* spp. in El-Dakahlia (1.8%) was higher than in El-Gharbia (1.1%) and Damietta (0.8%). In contrast, the infection rate of *G. duodenalis* was higher in El-Dakahlia (11.4%) and El-Gharbia (12.7%) than in Damietta (8.9%; Table 1).

There was a significant negative correlation between age and diarrhea (correlation coefficient was -0.115 and -0.127. by Kendall’s tau_b and Spearman’s rho tests, respectively; *P =* 0.002 in both tests).

### *Cryptosporidium* species and subtypes

The RFLP analysis of the *SSU rRNA* PCR products identified the presence of *C. hominis* in five specimens and *C. parvum* in three specimens (Table 2). Three subtype families were identified within *C. hominis* and *C. parvum* each by *gp60* sequence analysis. The *C. hominis* subtypes families included Ib (in two specimens), Id (in two specimens) and If (in one specimen), while the *C. parvum* subtypes families included IIa, IIc, and IId (in one specimen each). There were two subtypes (IdA17 and IdA24) in the subtype family Id and one subtype each in subtype families Ib (IbA6G3 in two specimens) and If (IfA14G1R5 in one specimen). The *C. parvum* subtypes detected included IIaA15G2R1, IIdA20G1 and IIcA5G3a (in one specimen each).

**Table 2:** Characteristics of eight *Cryptosporidium-*positive children

*Cryptosporidium* spp.	Subtypes	Age (years)	Gender	Diarrhea occurrence	Abdominal pain occurrence	Animal contact	Residency
*Cryptosporidium hominis*	IbA6G3^a^	4	Female	No	Yes	No	Urban
	IbA6G3^a^	4	Male	No	Yes	No	Urban
	IdA17	2	Male	No	Yes	No	Urban
	IdA24	5	Male	No	Yes	No	Rural
	IfA14G1R5	3.5	Female	No	No	No	Rural
*Cryptosporidium parvum*	IIaA15G2R1	5.5	Male	Yes	Yes	Yes	Rural
	IIdA20G1^a^	3	Male	Yes	Yes	Yes	Rural
	IIcA5G3a	6	Female	No	Yes	Yes	Rural

^a^New subtype identified in humans in Egypt

### *Giardia duodenalis* genotypes and subtypes

Of the 66 *G. duodenalis*-positive specimens, 56 were positive in *tpi* PCR, 48 in *gdh* PCR, and 55 in *bg* PCR. Among them, 31 (47.0%) had assemblage A and 34 (51.5%) had assemblage B, with one specimen (1.5%) being positive for both assemblages A and B (Table 3). The latter was indicated by the identification of assemblage B at the *tpi* and *gdh* loci but assemblage A at the *bg* locus. There were mostly no double peaks in the chromatograms generated from the study. Assemblage A was identified in 28 specimens based on *tpi* and *bg* sequence analyses but in 25 specimens by *gdh* sequence analysis. In contrast, assemblage B was found in 28, 23 and 27 specimens at the *tpi*, *gdh* and *bg* loci, respectively (Table 3). The relative distribution of *G. duodenalis* assemblages A and B was similar among three provinces (Table 4); assemblage A was detected in 14, 11 and 6 specimens from El-Dakahlia, El-Gharbia and Damietta provinces, respectively, whereas, assemblage B was detected in 16, 13 and 5 specimens, respectively.

**Table 3:** Distribution of *G. duodenalis* assemblages in children from different kindergartens at the *tpi*, *gdh* and *bg* loci

Study area^a^		No. of samples	No. of positive (%)	Number of positive					
				*tpi*		*gdh*		*bg*	
				Assemblage A (*n*)	Assemblage B (*n*)	Assemblage A (*n*)	Assemblage B (*n*)	Assemblage A (*n*)	Assemblage B (*n*)
El-Dakahlia	K1	34	2 (5.9)	0	2	0	1	0	1
	K2	39	8 (20.5)	5	3	3	3	4	3
	K3	33	3 (9.1)	2	0	3	0	2	0
	K4	31	3 (9.7)	1	2	1	2	1	2
	K5	22	2 (9.1)	2	0	1	0	1	0
	K6	35	6 (17.1)	2	4	2	3	3	2
	K7	37	2 (5.4)	0	2	0	2	0	2
	K8	41	5 (12.2)	1	3	1	3	1	4
El-Gharbia	K1	27	3 (11.1)	2	1	1	1	1	1
	K2	30	5 (16.7)	2	2	2	2	2	3
	K3	34	5 (14.7)	4	1	3	1	4	1
	K4	29	2 (6.9)	0	2	0	1	0	1
	K5	32	4 (12.5)	1	2	1	0	1	2
	K6	37	5 (13.5)	2	2	2	1	2	2
Damietta	K1	22	3 (13.6)	1	0	2	1	2	1
	K2	28	4 (14.3)	1	1	1	2	2	1
	K3	38	4 (10.5)	2	1	2	0	2	1
	K4	36	0 (0.0)	0	0	0	0	0	0
Total		585	66 (11.3)	28	28	25	23	28	27

^a^K, kindergarten

**Table 4:** Distribution of *Cryptosporidium* species and subtypes and *Giardia duodenalis* assemblages by locality

Province	*Cryptosporidium* spp.		*Giardia duodenalis*		
	Species (*n*)	Subtypes (*n*)	Assemblage A	Assemblage B	Assemblages A+B
El-Dakahlia	*C. parvum* (2)	IIaA15G2R1 (1); IIcA5G3a (1)	14	16	1
	*C. hominis* (3)	IbA6G3 (1); IdA17 (1); IdA24 (1)			
El-Gharbia	*C. parvum* (1)	IIdA20G1 (1)	11	13	0
	*C. hominis* (1)	IbA6G3 (1)			
Damietta	*C. hominis* (1)	IfA14G1R5 (1)	6	5	0
Total	*C. parvum* (3)	IIdA20G1 (1); IIaA15G2R1 (1); IIcA5G3a (1)	31/66 (47.0%)	34/66 (51.5%)	1/66 (1.5%)
	*C. hominis* (5)	IbA6G3 (2); IdA17 (1); IdA24 (1); IfA14G1R5 (1)			

### Multilocus genotypes (MLGs) of *G. duodenalis*

Sequence analysis of the three genetic loci showed only limited genetic diversity in assemblage A. All identified subtypes were belonged to sub-assemblage AII. Therefore, at the *tpi* locus, all assemblage A sequences were identical to the A2 subtype sequence (U57897) in GenBank (Table 5). Similarly, at the *gdh* locus, all 25 assemblage A sequences obtained were identical to the A2 subtype sequence (AY178737) in GenBank, while at the *bg* locus, 22 were identical to the A3 subtype (AY072724), 4 were identical to the A2 subtype (AY072723), and 2 belonged to a new subtype A9 (MG746615). Among the assemblage A specimens, 4 and 18 specimens had MLGs AII-1 and AII-9, respectively. In addition, one new MLG AII-15 was identified in one specimen (Table 5). In contrast, each of the 20 MLGs of assemblage B was identified in only one specimen.

**Table 5:** Multilocus sequence types of *Giardia duodenalis* assemblage A in children, Egypt

MLGs	Sequence type (GenBank ID)			No. positive	Specimen ID
	*tpi*	*gdh*	*bg*		
AII-1	A2 ( U57897)	A2 (AY178737)	A2 (FJ560582)	4	43567, 43664, 43968, 44106
AII-9	A2 (U57897)	A2 (AY178737)	A3 (AY072724)	18	43509, 43524, 43547, 43574,43581,43608, 43618, 43632, 43700, 43899, 43907, 43956, 44038, 44042, 44046, 44069, 44116, 44170
AII-15	A2 ( U57897)	A2 (AY178737)	A9^a^ (MG746615)	1	43969
–	B^a^ (MG787952)	B^a^ (MG746609)	A3 (AY072724)	1	43642
–	A2 (U57897)	–	A3 (AY072724)	1	43532
–	A2 (U57897)	–	A9^a^ (MG746615)	1	43936
–	–	A2 (AY178737)	A3 (AY072724)	1	44067
–	A2 (U57897)	–	–	3	43503, 43607, 43894
–	–	A2 (AY178737)	–	1	43569
–	–	–	A3 (AY072724)	1	44095

^a^New sequence type identified in the study

Much higher genetic diversity was seen in assemblage B (Additional file 1: Table S1). Of the 28 specimens that were positive for assemblage B at the *tpi* locus, 14 had generated sequences identical to either KX668322 (*n* = 3), JF918523 (*n* = 2), KT948107 (*n* = 2), KT948111 (*n* = 2), AB781127 (*n* = 1), AY368163 (*n* = 1), JF918519 (*n* = 1), KY696816 (*n* = 1) or KX468984 (*n* = 1), while 14 specimens generated sequences of one of the 10 new types (MG787950–MG787959). Similarly, of the 23 specimens that were positive for assemblage B at the *gdh* locus, 14 had sequences identical to either KY696804 (*n* = 4), KM190714 (*n* = 3), KP687771 (*n* = 3), U362955 (*n* = 2), EF507654 (*n* = 1) or KP687770 (*n* = 1), while the remaining nine specimens produced sequences of one of the eight new types (MG746604–MG746611). At the *bg* locus, 24 specimens generated sequences identical to either KU504732 (*n* = 6), KY696836 (*n* = 5), JF918485 (*n* = 3), KU504720 (*n* = 2), KU504707 (*n* = 2), MF169196 (*n* = 2), AB480877 (*n* = 1), KT948086 (*n* = 1), KU504731 (*n* = 1) or KY483962 (*n* = 1), whereas three specimens yielded sequences that belonged to one of the three new subtypes (MG746612–MG746614). Altogether, 44 specimens were successfully subtyped at all three genetic loci, forming 3 MLGs of assemblage A and 20 MLGs of assemblage B.

## Discussion

In the present study, the overall infection rates of *Cryptosporidium* spp. and *G. duodenalis* in children were 1.4 and 11.3%, respectively. Earlier studies based on microscopy had recorded 5.6–60.2% and 17.6–25.0% infection rates of *Cryptosporidium* spp. and *G. duodenalis* in Egyptian children, respectively [24–27]. A previous molecular analysis of fecal specimens from Egyptian children produced 49.1% and 21% infection rate for *Cryptosporidium* spp. and *G. duodenalis*, respectively [13, 17]. In the neighboring Lebanon, infection rates of 10.4% and 28.5% were reported in school children for *Cryptosporidium* spp. and *G. duodenalis*, respectively [28]. Similar low *Cryptosporidium* occurrence (1.6–2.0%) was observed in children in China [29, 30]. The low occurrence of *Cryptosporidium* spp. in this study might be due to the older age of children enrolled in this study. In developing countries, children under two years have the highest occurrence of *Cryptosporidium* spp. [4, 31]*.* In addition, children participating in the study were healthy kindergartners rather than in-patients and outpatients in most previous studies. As expected, children with diarrhea had higher occurrence of both *Cryptosporidium* spp. and *G. duodenalis* in this and earlier studies [28]. These are also supported by results of the nonparametric analysis of the negative correlation between age and occurrence diarrhea in this study.

In our study, we identified only *C. hominis* and *C. parvum* in children. This is similar to results of other studies in Egypt [13, 14, 32]. Moreover, the more common occurrence of *C. hominis* in children in this and other African studies suggests that anthroponotic transmission is important in cryptosporidiosis epidemiology in this area, although the occurrence of zoonotic infections could not be fully excluded [13–15, 28, 32–35]. This is also supported by the identification of IIcA5G3a in *C. parvum*, which is considered a human-adapted *C. parvum* subtype [8]. In contrast, previous studies in the neighboring Mideast countries had shown a dominance of the zoonotic IIa and IId subtypes of *C. parvum* in children, which were only identified in two of the eight cryptosporidiosis cases in this study [36–40]. The insignificant associations between cryptosporidiosis occurrence and animal contact or rural residency in this study also support the importance of anthroponotic transmission in *Cryptosporidium* spp. in Egyptian children.

Although *Cryptosporidium* spp. were detected in only a few specimens in the study, we recorded seven subtypes in six families, including Ib, Id and If subtype families of *C. hominis* and IIa, IIc, and IId subtype families of *C. parvum*. This indicates that the transmission of *Cryptosporidium* in the study area is intensive. It has been reported that subtype families Ia, Ib, Id and Ie are common in children in developing countries [8, 31]. Nevertheless, the IbA6G3, IdA17, IdA24, and IfA14G1R5 identified in this study are rare subtypes within these common *C. hominis* subtype families [8, 31], indicating that *C. hominis* transmission in Egypt is probably autochthonous in nature.

The genotypes (assemblages of similar sequence types identified by multilocus molecular characterization) of *G. duodenalis* in infected children from the three provinces in this study belonged to assemblages A and B. This agrees with the findings of a recent study of *G. duodenalis* in children in Egypt [18]. The assemblages E and C reported in a few Egyptian children in previous studies [16, 17] were not detected in the present study. The equal occurrence of assemblages A and B in the present study is in discordance with observations in previous Egyptian studies, which showed a dominance of assemblage B in children [16–18]. Globally, assemblage B is more common than assemblage A in humans [7]. As assemblage B is much less frequently detected in animals [2], *G. duodenalis* transmission in Egyptian children appears to be mostly anthroponotic. This is also supported by the identification of assemblage A isolates in the study as the sub-assemblage AII, which is preferentially found in humans [7].

In this study, a much higher genetic diversity was observed in assemblage B than in assemblage A. Similar observations were made in previous studies [2]. This could be due to the more frequent occurrence of genetic recombination among assemblage A isolates, as assemblage B is known to have much higher allelic sequence heterozygosity than assemblage A. The existence of highly genetic variations among isolates of assemblage B has led to the inability of categorizing assemblage B isolates into well-defined specific sub-assemblages [9]. Comparative genomics rather than current MLG analysis might be needed for better characterization of assemblage B isolates [41].

## Conclusions

Giardiasis is apparently common, and cryptosporidiosis remains to be a problem in kindergarten age children in Egypt. The dominance of *C. hominis* and common occurrence of *G. duodenalis* assemblage B and sub-assemblage AII in clinical specimens showcases the important role of anthroponotic transmission in disease epidemiology, although the occurrence of zoonotic infections could not be totally ruled out. Improved sanitation and hygiene and other intervention measures such as better health communication and the provision of clean and safe drinking water should be implemented to reduce the occurrence of cryptosporidiosis and giardiasis and minimize the impact of diarrhea on pediatric health in the country.

## Additional file

Additional file 1: **Table S1.** Specimens from kindergarten-age children in Egypt that were positive for *Giardia duodenalis* assemblage B at the *tpi*, *gdh* and *bg* loci. (DOCX 16 kb)

## Abbreviations

*bg*: β-giardin
*gdh*: Glutamate dehydrogenase
MLGs: Multilocus genotypes
RFLP: Restriction fragment length polymorphism
*SSU rRNA*: Small subunit ribosomal ribonucleic acid
*tpi*: Triose phosphate isomerase
