# Supplementary material for: Molecular characterization of Cryptosporidium spp. and Giardia duodenalis in children in Egypt
Source: Parasit Vectors. 2018 Jul 11;11:403. doi: 10.1186/s13071-018-2981-7 (PMC6042380; doi:10.1186/s13071-018-2981-7)
Supplement: Supplementary file 1 — Table S1. Specimens from kindergarten-age children in Egypt that were positive for Giardia duodenalis assemblage B at the tpi, gdh and bg loci. (DOCX 16 kb) [file 13071_2018_2981_MOESM1_ESM.docx]

**Additional file 1: Table S1.** Specimens from kindergarten-age children in Egypt that were positive for *Giardia duodenalis* assemblage B at the *tpi*, *gdh*, and *bg* loci

| **Specimen ID** | **Sequence type (GenBank ID)** | | | **No. positive** | **MLGs** |
| --- | --- | --- | --- | --- | --- |
|  | ***tpi*** | ***gdh*** | ***bg*** |  |  |
| 43488 | B (KT948107) | B^a^ (MG746604) | B (KY696836) | 1 | MLG B1 |
| 43504 | B (JF918519) | B^a^ (MG746605) | B1 (KU504731) | 1 | MLG B2 |
| 43531 | B (KT948111) | B^a^ (MG746606) | B (KU504720) | 1 | MLG B3 |
| 43535 | B (KX668322) | B^a^ (MG746607) | B (KU504707) | 1 | MLG B4 |
| 43597 | B (KT948111) | B (EU362955) | B (JF918485) | 1 | MLG B5 |
| 43605 | B^a^ (MG787950) | B^a^ (MG746607) | B1 (KY696836) | 1 | MLG B6 |
| 43615 | B^a^ (MG787951) | B^a^ (MG746608) | B1 (KY696836) | 1 | MLG B7 |
| 43638 | B (KX668322) | B (KY696804) | B (KU504732) | 1 | MLG B8 |
| 43651 | B (KY696816) | B (KY696804) | B1 (KY696836) | 1 | MLG B9 |
| 43652 | B^a^ (MG787953) | B^a^ (MG746610) | B (AB480877) | 1 | MLG B10 |
| 43653 | B^a^ (MG787954) | B (EF507654) | B1^a^ (MG746612) | 1 | MLG B11 |
| 43671 | B (KX468984) | B (KM190714) | B (KU504732) | 1 | MLG B12 |
| 43690 | B^a^ (MG787955) | B (KM190714) | B^a^ (MG746614) | 1 | MLG B13 |
| 43856 | B5^a^ (MG787956) | B^a^ (MG746611) | B (KU504732) | 1 | MLG B14 |
| 43897 | B (JF918523) | B (KP687771) | B (KY483962) | 1 | MLG B15 |
| 43901 | B (JF918523) | B (KM190714)) | B1 (MF169196) | 1 | MLG B16 |
| 43927 | B^a^ (MG787956) | B (KP687771) | B1 (KY696836) | 1 | MLG B17 |
| 43995 | B^a^ (MG787950) | B (KP687770) | B1 (KT948086) | 1 | MLG B18 |
| 43059 | B^a^ (MG787958) | B (KY696804) | B (KU504732) | 1 | MLG B19 |
| 44084 | B^a^ (MG787959) | B (KY696804) | B (KU504732) | 1 | MLG B20 |
| 43642 | B^a^ (MG787952) | B^a^ (MG746609) | A3 (KY499042) | 1 | MLG A+B |
| 44023 | B (AB781127) | – | B1 (MF169196) | – | – |
| 44062 | – | B (KP687771) | B (KU504707) | – | – |
| 43641 | B (KX668322) | – | – | – | – |
| 43972 | B^a^ (MG787950) | – | – | – | – |
| 44058 | B^a^ (MG787958) | – | – | – | – |
| 44001 | B^a^ (MG787957) | – | – | – | – |
| 44139 | B (AY368163) | – | – | – | – |
| 43489 | B (KT948107) | – | – | – | – |
| 44092 | – | B (EU362955) | – | – | – |
| 43909, 44041 | – | – | B (JF918485) | – | – |
| 44054 | – | – | B (KU504720) | – | – |
| 44124 | – | – | B (KU504732) | – | – |
| 43684 | – | – | B3^a^ (MG746613) | – | – |

^a^ New sequence type identified in the study.
